# CD82 Suppresses ADAM17-Dependent E-Cadherin Cleavage and Cell Migration in Prostate Cancer

**DOI:** 10.1155/2020/8899924

**Published:** 2020-11-01

**Authors:** Zhenkun Ma, Ye Gao, Wei Liu, Long Zheng, Ben Jin, Bei Duan, Hongjun Xie, Peng Guo, Jin Zeng, Ke Wang, Shan Xu, Xinyang Wang, Dalin He, Lei Li

**Affiliations:** ^1^Department of Urology, The First Affiliated Hospital of Xi'an Jiaotong University, 277 Yanta West Road, Xi'an 710061, China; ^2^Department of Emergency, The First Affiliated Hospital of Xi'an Jiaotong University, 277 Yanta West Road, Xi'an 710061, China; ^3^Department of Obstetrics and Gynecology, The Second Affiliated Hospital of Shaanxi University of Chinese Medicine, 5 Weiyang West Road, Xianyang 712000, China

## Abstract

CD82 acts as a tumor suppressor in a series of steps in malignant progression. Here, we identified a novel function of CD82 on posttranslational regulating E-cadherin in prostate cancer. In our study, the declined expression of CD82 was verified in prostate cancer tissues and cell lines compared with normal tissue and cell lines. Functionally, CD82 inhibited cell migration and E-cadherin cleavage from the cell membrane in prostate cancer cell. Further study proved that a disintegrin and metalloproteinase ADAM17 as an executor of E-cadherin cleavage mediated the inhibitory regulation of CD82 in E-cadherin shedding in prostate cancer. Specifically, CD82 interacted with ADAM17 and inhibited its metalloprotease activity, which led to the descent of E-cadherin shedding. These results show a nuanced but important role of CD82 in nontranscriptional regulation of E-cadherin, which may help to understand the intricate regulation of dysfunctional adhesion molecule in cancer progression.

## 1. Introduction

According to the recent cancer statistics, prostate cancer was the second most frequent cancer and the fifth leading cause of cancer death in men worldwide [1]. The metastasis marks the lethal progression and recurrence of tumor. However, the exact mechanism involved has not been illuminated clearly.

As a key adhesive molecule in the prevention of tumor progression, E-cadherin undergoes a series of negative regulations in multiple tumors, including mutations [2, 3], epigenetic silencing [4, 5], transcriptional regulation [6], and endocytosis [7]. Besides those above, as a membrane protein, E-cadherin was also processed through proteolytic modification.

CD82 was found to inhibit tumor metastasis [8] as well as cell death [9], senescence [10], angiogenesis [11], and so on. It belongs to the superfamily of tetraspanins, which interact with adjacent membrane proteins and cytoplastic factors to regulate their functions such as integrins and receptor tyrosine kinase (RTK) [12, 13]. Abe et al. indicated that CD82 prevents cancer cell disseminating from primary site via stabilizing the E-cadherin/*β*-catenin complex, which is attributed to the reduced tyrosine phosphorylation of *β*-catenin by CD82 [14]. However, whether CD82 participates in regulating E-cadherin still needs to be elucidated.

Since CD82 is located through the cell membrane and most of its functions resorted to the tetraspanin-enriched membrane microdomains (TEM) [15], we focused on the proteolytic modification to unveil the function of CD82 on posttranslational regulation of E-cadherin, which may improve our understanding of the metastatic suppressor role of CD82 and get much more details on the metastasis progress of prostate cancer.

## 2. Methods

### 2.1. Cell Culture and Reagents

Human prostate cancer cell lines LNCaP, C4-2, C4-2B, CWR22Rv1, PC-3, DU145, and human benign prostatic hyperplasia cell (BPH1) and normal prostatic epithelial cell (RWPE1) were purchased from American Type Culture Collection (Manassas, VA, USA) and cultured in RPMI-1640 medium supplemented with 10% fetal bovine serum (Gibco, NY, USA) at 37°C, in humidified air containing 5% of CO_2_. MTT (3-(4,5-dimethylthiazol-2-yl)-2,5-diphenyltetrazolium bromide) was purchased from Sigma-Aldrich Co. (MO, USA). Rabbit anti-CD82 (Cat. No. ab109529) and rabbit anti-ADAM17 (Cat. No. ab173579) monoclonal antibodies were purchased from Abcam (MA, USA), and mouse anticytoplasmic fragments of E-cadherin monoclonal antibody were from BD Biosciences (Cat. No. 610181, CA, USA), and rabbit anti-HA (Cat. No. 3724S) and rabbit anti-Flag (Cat. No. 14793S) monoclonal antibodies were from Cell Signaling Technology, Inc. (MA, USA). Mouse anti-Flag monoclonal antibody (Cat. No. F1804) was from Sigma-Aldrich Co. (MO, USA). Mouse anti-HA monoclonal antibody (Cat. No. 26183) was from Thermo Fisher Scientific Inc. (MA, USA). Mouse anti-GAPDH monoclonal antibody was purchased from Kangchen Bio-tech Inc. (Cat. No. KC-5G4, Shanghai, China).

### 2.2. Small Interfering RNA Transfection

Small interfering RNAs were designed and synthesized by GenePharma (Shanghai, China) for CD82 and RiboBio (Guangzhou, China) for ADAMs. When cells achieved 30-50% confluency, siRNAs were transfected with X-tremeGENE siRNA Transfection Reagent (Roche, Germany) for 48-72 hours and harvested for the next experiments. The siRNA sequences are shown in Supplementary Table 1.

### 2.3. Plasmid Transfection

CD82 cDNA was cloned into pcDNA3.1 vector. When cells achieved 70-80% confluency, pcDNA3.1 vector and pcDNA3.1-CD82 plasmids were transfected with X-tremeGENE HP DNA Transfection Reagent (Roche, Germany). Cells continued to be cultured for another 72 hours and harvested for the next experiments.

### 2.4. Lentivirus Transfection

The CD82 overexpression/shRNA plasmids containing lentiviruses were constructed by GenePharma (Shanghai, China). Cells were transfected with lentivirus in the presence of 5 *μ*g/ml polybrene and selected by puromycin to create stable cell lines.

### 2.5. Migration Assay

Cells were prepared and suspended in serum-free media and seeded into the upper chambers of the transwell system with 6‐10 × 10^4^ cells per well; 800 *μ*l medium containing 10% FBS was added into the lower chambers. After a 24-hour culture, the penetrated cells were fixed in 10% formalin for 10 minutes and stained with 0.1% crystal violet solution for 5 minutes. Cell numbers were calculated under the view of a microscope. The experiments were performed in triplicate.

### 2.6. Cell Viability Assay

Cell viabilities were detected by tetrazolium-based assay. Cells of each group were seeded in a 96-well plate and incubated for the indicated times (0 h, 24 h, 48 h, and 72 h). The media were removed, and cells were incubated with 0.5 mg/ml MTT for 4 hours. After the formazan products were fully resolved with DMSO (150 *μ*l/well), the OD value of each well was measured by a microplate reader at a wavelength of 490 nm. The experiments were performed in triplicate.

### 2.7. Immunoblot Analysis

Cell lysates were prepared in lysis buffer (10 mM of Tris-HCl (pH of 7.4), 150 mM of NaCl, 0.1% of SDS, 1 mM of EDTA, 1 mM of EGTA, 0.3 mM of PMSF, 0.2 mM of sodium orthovanadate, 1% of NP-40, 10 mg/ml of leupeptin, and 10 mg/ml of aprotinin). The samples of protein were separated in SDS-PAGE on 10% or 12% Tris-Glycine gels and transferred onto nitrocellulose membranes by Western blot. The membranes were blocked with 5% skim milk in TBS for 1 hour at room temperature and probed with primary antibodies overnight at 4°C followed by secondary antibody incubation for 1 hour at room temperature. The protein expressions were visualized with ECL detection system or Odyssey Infrared Imaging System (LI-COR Biosciences). The ratio of CTF/full-length E-cadherin protein expressions was analyzed with band densities by ImageJ software (version 1.44p).

### 2.8. Reverse Transcription and Real-Time PCR

Total RNA was isolated with RNAfast 200 reagents (FASTAGEN, Shanghai, China) following the instruction and quantitated by absorbance at 260 nm. 0.5 *μ*g RNA sample was used for reverse transcription with PrimeScript™ RT Master Mix (Takara, Dalian, China) according to the manufacturer's instruction; quantitative PCR was performed with SYBR Green PCR Master Mix (Takara, Dalian, China), and GAPDH mRNA was used as internal control. The primer sequences are listed in Supplementary Table 2.

### 2.9. Immunohistochemistry

The prostate cancer tissue array slides (HProA100PG01) were purchased from Shanghai Outdo Biotech (Shanghai, China). The immunohistochemistry staining was performed with EnVision™ System (DAKO, Carpinteria, CA, USA). The slides were deparaffinized and rehydrated followed by 5 minutes of antigen retrieval, 10 minutes of endogenous enzyme block, and overnight incubation with primary antibody at 4°C. Then, the slides were incubated with EnVision-HRP secondary antibody for 1 hour, and the signal was detected by diaminobenzidine (DAB) followed by hematoxylin counterstaining. The results were observed by a microscope.

### 2.10. Immunoprecipitation

Cells were prepared in lysis buffer (50 mM Tris-HCl, pH 7.4, with 150 mM NaCl, 1 mM EDTA, and 1% Triton X-100, adding 1 mM PMSF before use), and the supernatant was collected by centrifugation (at 4°C, 8000 rpm, 15 minutes). 200 *μ*l cell lysate at 1 mg/ml was added with primary antibody and incubated with gentle rocking overnight at 4°C. Either protein A or G agarose beads were added into the lysis for another incubation with gentle rocking for 4–6 hours at 4°C. The slurries were centrifuged for 1 minute and washed twice with lysis buffer. The beads were heated to 95–100°C for 5 minutes and centrifuged for 1 minute, and the supernatant was collected for Western blot analysis.

### 2.11. Immunofluorescence Staining

Cells were fixed in 4% paraformaldehyde for 15 minutes and permeabilized in 0.5% Triton X-100 for 5 minutes. Cells were then blocked in 5% bovine serum albumin for 1 hour and incubated with primary antibodies overnight at 4°C. The cells were washed and incubated with fluorescent secondary antibodies followed by DAPI staining. The results were observed by a fluorescence microscope.

### 2.12. Analysis of TACE Activities

Cells were lysed with a weak lysis buffer (50 mM Tris-HCl, pH 7.4, 150 mM NaCl, 1 mM EDTA, and 1% Triton X-100 buffer). The protein extract of 60 mg and 10 mM TACE Substrate (Enzo Life Sciences, USA) was mixed in 150 *μ*l 50 mM Tris-HCl, pH 7.4, 25 mM NaCl and 4% glycerol buffer and incubated at 37°C in the dark. The fluorescence was measured every 5 minutes for 20 minutes (excitation: 320 nm; emission: 420 nm). A unit of TACE activity is the amount of active enzyme necessary to produce an increase in 1 fluorescence unit in the luminescence spectrophotometer. Subsequently, the protein concentration of the cell lysates was determined and the results presented as units of TACE activity/h/mg of protein.

### 2.13. Statistical Analysis

All the statistical analyses were performed by GraphPad Prism (version 5.0) software. Student's *t* test was used for two groups' comparison, and one-way ANOVA test was used for multiple groups' comparison follow by Dunnett *t* test. *P* < 0.05 was considered statistically significant.

## 3. Result

### 3.1. CD82 Is Suppressed in Prostate Cancer Compared with Normal Tissue

**Figure 1:**
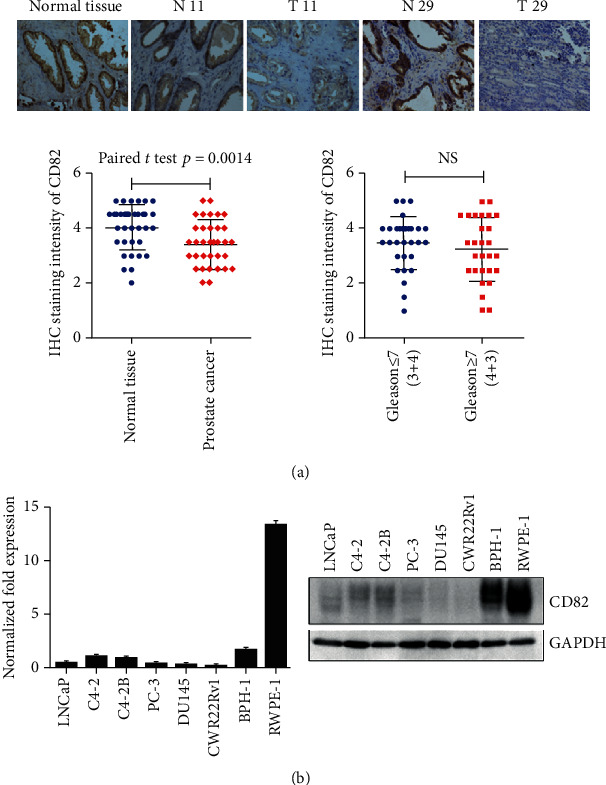
The expression of CD82 in PCa tissues and cell lines. (a) Immunohistochemical assay was applied to detect the expressions of CD82 in normal prostate tissues, prostate cancers, and matched adjacent normal tissues. Quantification of CD82 staining was shown in cancer and matched adjacent tissue and in cancers with different Gleason scores (N: matched adjacent normal tissue, T: prostate cancer). (b) Real-time PCR (left) and Western blot (right) showed the expressions of CD82 in prostate normal epithelia cells RWPE-1, benign prostatic hyperplasia cell BPH-1, and various PCa cell lines. Error bars indicate s.d. from at least two technical replicates.

The decreased expression of CD82 was detected in prostate cancer tissue by immunohistochemical assay compared with the matched adjacent normal tissue. However, no significant distinction had been found between the prostate cancer (PCa) with high Gleason score [≥7(4 + 3)] and the PCa with low Gleason score [≤7(3 + 4)] (Figure 1(a)). CD82 expressions were obviously decreased in prostate cancer cells (LNCaP, C4-2, C4-2B, PC-3, DU145, and CWR22Rv1) compared with prostate normal epithelia cell RWPE-1 and benign prostatic hyperplasia cell BPH-1 by real-time PCR and Western blot (Figure 1(b)).

### 3.2. CD82 Reduces the Migrating Properties of Prostate Cancer Cells

**Figure 2:**
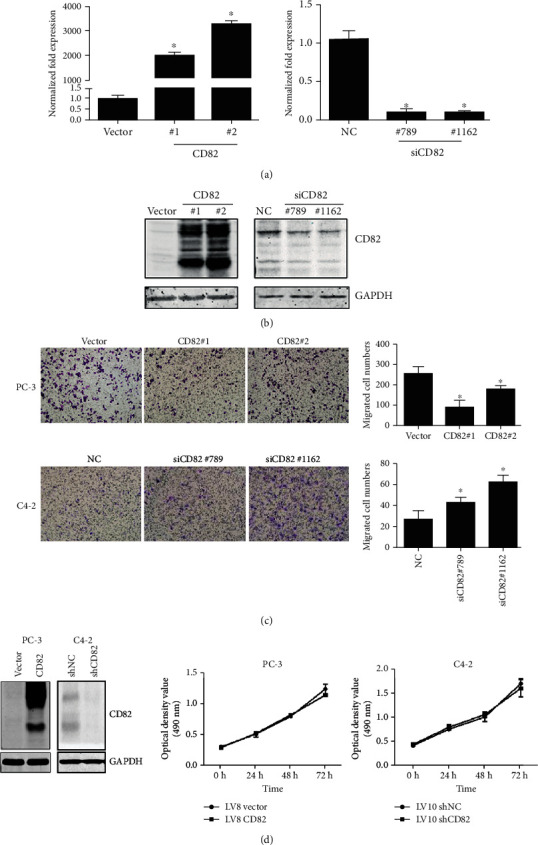
The effects of CD82 on PCa cell migration. PC-3 was transfected with control vector, pcDNA3.0-CD82#1, and pcDNA3.0-CD82#2, while C4-2 was transfected with scramble negative control, siRNA-CD82#789, and siRNA-CD82#1162, and the expressions of CD82 were determined by (a) real-time PCR and (b) Western blot. (c) The properties of cell migration were identified by transwell assay with quantification results. (d) Cell growth properties were detected by tetrazolium-based assay in PC-3 with CD82 overexpression and C4-2 with CD82 knockdown. ^∗^*P* < 0.05. Error bars indicate s.d. from at least two technical replicates.

Transient overexpressing CD82 in PC-3 cells significantly reduced the migrating abilities *in vitro*, while knocking down of CD82 with small interfering RNA in C4-2 cells enhanced the cell migration (Figures 2(a)–2(c)). Consistent with the results above, stable clones established by lentivirus systems showed the suppressor role of CD82 in migration as well (Supplementary Figure 1 (a)). Meanwhile, we observed that CD82 had no effect on prostate cancer cell growth (Figure 2(d)).

### 3.3. CD82 Inhibits the Cleavage of E-Cadherin

**Figure 3:**
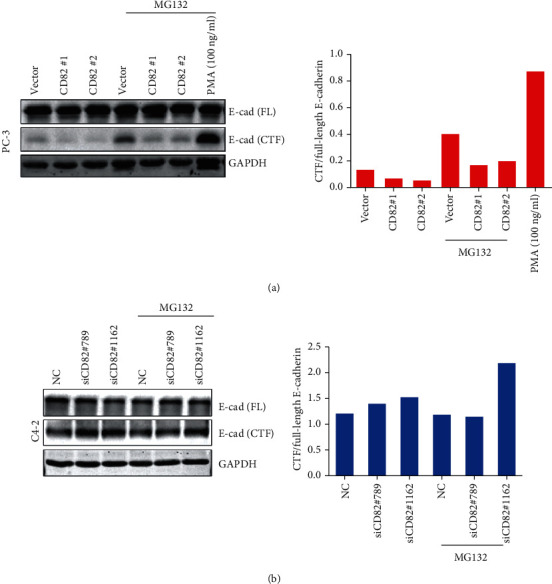
CD82 inhibited the cleavage of E-cadherin in PCa cells. In (a) PC-3 with CD82 overexpression and (b) C4-2 with CD82 knockdown, Western blot detected the full-length E-cadherin (FL) and cytoplasmic E-cadherin fragments (CTF) (left), and the ratios of shedding fragments to full-length E-cadherin were quantified (right).

E-cadherin is considered to be a crucial adhesion molecule involved in a series of steps in cancer metastasis. Regulation of E-cadherin might effectively influence the cancer progression. We found that the cytoplasmic fragments of E-cadherin (C-terminal fragment (CTF)) were dramatically decreased with CD82 overexpression in PC-3 cells (Figure 3(a)). In contrast, the cytoplasmic fragments of E-cadherin were increased in C4-2 cells with knockdown of CD82 (Figure 3(b)). Meanwhile, the proteasome inhibitor MG132 (10 *μ*M, 8 hours) enhanced the accumulation of cytoplasmic fragment in PC-3 cells. PMA (phorbol 12-myristate 13-acetate) was a PKC stimulator that was used as a positive control for E-cadherin shedding. Stable clones of CD82-overexpressing CWR22Rv1 cells also supported the conclusion above (Supplementary Figure 1(b)).

### 3.4. Knocking Down of ADAM17 Reverses the Increasing Cleavage of E-Cadherin Caused by CD82 Reduction

**Figure 4:**
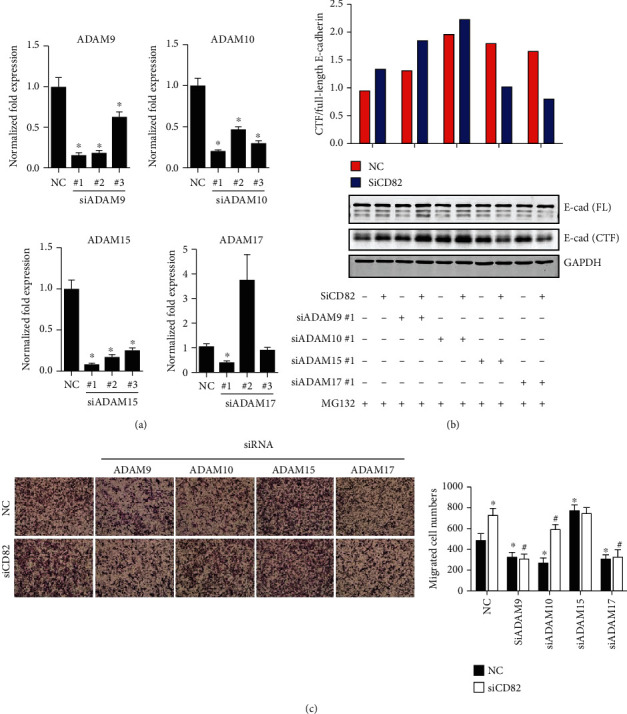
The silence of ADAM17 reversed the shedding of E-cadherin caused by knocking down of CD82. (a) Small interfering RNAs were used to silence the expressions of ADAMs, and real-time PCR was performed to verify the efficiency of siRNAs. (b) C4-2 cells were cotransfected with siRNA against both CD82 and ADAMs, and cytoplasmic fragments of E-cadherin were detected by Western blot, and the ratios of cytoplasmic E-cadherin fragments (CTF) to full length were quantified. (c) The cell migrating activities were analyzed by transwell assay. ^∗^*P* < 0.05. Error bars indicate s.d. from at least two technical replicates.

A disintegrin and metalloprotease (ADAM) family promotes tumor progression potentially due to its extracellular cleaving substrate properties. To explore the mechanism referring to CD82-mediated E-cadherin shedding, we reviewed the related researches and screened four ADAM candidates which had been reported as protease of cadherin (not only E-cadherin) [16–19, 36]. Our results showed that only ADAM17 knockdown remarkably reversed both the increased shedding of E-cadherin (Figures 4(a) and 4(b)) and the enhanced cell migration (Figure 4(c)) by CD82 silencing the most among all four ADAMs, implying that the effects of CD82 in E-cadherin shedding in PCa cells might be mediated by ADAM17.

### 3.5. CD82 Interacts with ADAM17 and Diminishes Its Enzymatic Activity

**Figure 5:**
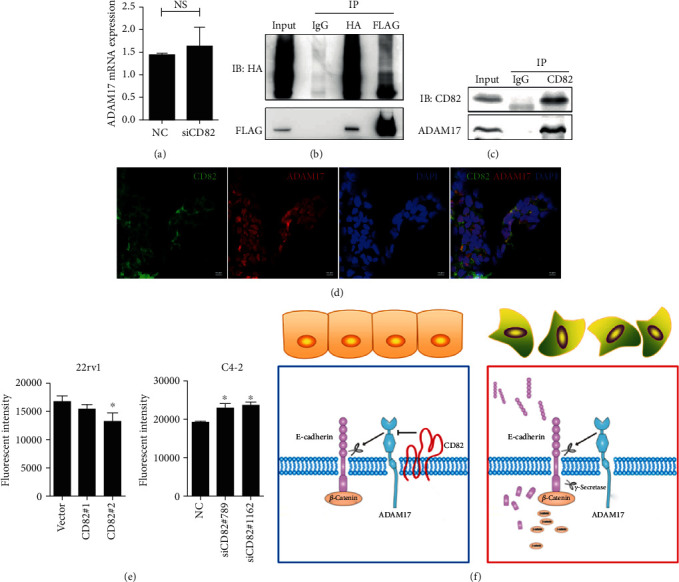
CD82 interacted with ADAM17 and inhibited the metalloprotease activity of ADAM17. (a) C4-2 cells were transfected with siRNA of CD82, and the transcriptional expressions of ADAM17 were measured by real-time PCR. (b) 293T cell transfected with pcDNA-2xHA-CD82 and pcDNA-2xFlag-ADAM17 after 72 hours was harvested for immunoprecipitation to identify the interaction of CD82 and ADAM17. (c) CWR22Rv1 cells reexpressing CD82 were applied to immunoprecipitation. (d) The coexpression of CD82 and ADAM17 was analyzed by immunofluorescence staining assay in CWR22Rv1 cells reexpressing CD82. (e) ADAM17 metalloprotease activities were monitored by fluorogenic substrate assay in PCa cells with diverse CD82 expressions. (f) Schematic depicting the effect of CD82 in E-cadherin shedding in this study. ^∗^*P* < 0.05. Error bars indicate s.d. from at least two technical replicates.

The next work was focused on how CD82 influenced the cleavage activity of ADAM17. As a transmembrane protein, CD82 usually interacts with other adjacent molecules on the membrane directly and influences their activities. Our results showed that CD82 had no effect on the expression of ADAM17 (Figure 5(a)). However, the immunoprecipitation results showed that CD82 could definitely interact with ADAM17 (Figures 5(b) and 5(c)), and the immunofluorescence assay also demonstrated an overlap between CD82 and ADAM17 in CWR22Rv1 cells (Figure 5(d)). Moreover, ADAM17 substrate assay verified that CD82 could withdraw the metalloprotease activity of ADAM17 (Figure 5(e)). A schematic was shown to depict the effect of CD82 in E-cadherin shedding in our study (Figure 5(f)).

## 4. Discussion

E-cadherin prevents cancer cells disseminating from primary lesion to distant organs via decreasing the motility and migratory and invasive properties. E-cadherin shedding yields an extracellular N-terminal 80 kDa fragment and an intracellular C-terminal 38 kDa fragment. The extracellular fragment also called soluble E-cadherin (sE-cad) was observed in the media of several types of cancer as a biomarker and reported to be associated with disease outcome and recurrence [20–23]. Meanwhile, the 38 kDa fragment was proved to further undergo an intracellular *γ*-secretase cleavage, and a 33 kDa fragment was shed into the cytoplasm and mediated cytoplasmic signal pathway. The cleavage of E-cadherin disassembled the E-cadherin/*β*-catenin complex and activated the Wnt/*β*-catenin pathway which contributed to tumor progression [24, 25]. A variety of stimuli triggered E-cadherin shedding including inflammatory cytokines and growth factors [26, 27]. Our study indicated that loss or reduction of CD82 in prostate cancer enhanced E-cadherin shedding from membrane, which might decrease the stabilization of E-cadherin/*β*-catenin complex. It provided a potential mechanism contributing to the metastasis of prostate cancer and a novel function of CD82 as a metastatic suppressor. As we mentioned before, E-cadherin forms a complex with *β*-catenin, and cleavage of E-cadherin may alter some cytoplasmic signal pathways through both yielding of intracellular C-terminal fragment and impairing the E-cadherin/*β*-catenin complex. Meanwhile, the sE-cadherin also plays a proper role in extracellular surrounding via autocrine or paracrine function. In prostate cancer, the serum sE-cad was increased in patients with metastatic disease compared with localized disease [28]. The sE-cad increased the motility and invasion of cancer cells as well as activation of EGFR signal and cell growth [19]. Therefore, the effects of CD82 in suppressing E-cadherin shedding from cell membrane may influence multiple steps of tumor progression through various pathways.

Similar to other tetraspanin family members, CD82 without self-enzymatic activity functions through interacting with other membrane molecules and regulating their expressions and activities. Therefore, E-cadherin may not be cleaved directly by CD82, but a mediator must get involved in the process. As is known, E-cadherin shedding requires *α*-secretase cleavage in the extracellular membrane which is catalyzed by various proteases such as a disintegrin and metalloproteinases (ADAMs) [16, 17]. ADAMs anchor through membrane and mediate the shedding and maturity of various membrane proteins such as HB-EGF, TNF*α*, notch, and cadherins and play roles in neuronal development, neurodegenerative disorders, and cancer progression. Cadherin shedding by ADAM mediates epithelial cell sorting and retinal ganglion cell differentiation, as well as cell-cell adhesion and *β*-catenin translocation [17, 18, 29, 30]. To testify whether E-cadherin shedding by CD82 relies on ADAMs, four ADAMs (9, 10, 15, and 17) were applied to our research. Integrating the results of E-cadherin shedding by Western blot and cell migrative abilities by transwell assay, we found that only knocking down of ADAM17 could both diminish the E-cadherin shedding and cell migration caused by silencing CD82. In addition to that, we discovered that knocking down of ADAM17 by siRNA could not reduce the E-cadherin shedding in C4-2 cells (CD82 positive) but it could significantly decrease its shedding in both PC-3 cells (CD82 negative) (Supplementary Figure 2) and CD82 knockdown C4-2 cells. This interesting data implied that the activity of ADAM17 may be mostly blocked by CD82 in prostate cancer cells. As an essential member of the ADAM family for the developmental and physiological processes according to studies in knockout mice, ADAM17 was originally found to be a protease processing of the precursor TNF*α* [31] and then discovered to be responsible for several substrates such as TNFR, L-selectin, TGF*α*, APP, IL-1R, VCAM-1, and EGF and its receptors [32–36]. Here, our results showed that E-cadherin may be another important substrate of ADAM17. It would be evidential to target ADAM17 as a potential treatment strategy for the metastatic prostate cancer. Actually, there had already been some inhibitors against ADAM17 explored as anti-inflammatory agents. Those drugs might be tested for their potential anticancer activities in our further study.

Our results indicated that CD82 could directly interact with ADAM17 and influence its protease activity, which refreshed our current knowledge about the mechanism of CD82 as a tumor metastasis suppressor. However, the exact mechanism how CD82 regulates the protease activity of ADAM17 still needs to be elucidated. Our further works may focus on that next. As ADAM17 affects a group of substrates which influence the signal transduction from extracellular surroundings into the cytoplasm and nuclei, CD82 is supposed to regulate the activities of the other substrates besides E-cadherin and play functions more widely. That is another interesting field we may concentrate our minds on in our further researches.

## Data Availability

All data generated or analyzed during this study are included in this published article.

## Supplementary Materials

Supplementary 1 Supplementary Table 1: siRNA sequences used in the experiments.

Supplementary 2 Supplementary Table 2: primer sequences used in real-time PCR in the experiments.
