# Supplementary material for: CD82 Suppresses ADAM17-Dependent E-Cadherin Cleavage and Cell Migration in Prostate Cancer
Source: Dis Markers. 2020 Nov 1;2020:8899924. doi: 10.1155/2020/8899924 (PMC7654213; doi:10.1155/2020/8899924)
Supplement: Supplementary 1 — Supplementary Table 1: siRNA sequences used in the experiments. [file 8899924.f1.docx]

Supplementary TABLE 2: Primer sequences used in real-time PCR in the experiments.

| Genes | Forward Primers | Reverse Primers |
| --- | --- | --- |
| CD82 | GCTCATTCGAGACTACAACAGC | GTGACCTCAGGGCGATTCA |
| ADAM9 | TCCATTGCTCTTAGCGACTGT | GGGGTTCAATCCCATAACTCG |
| ADAM10 | GAATTGCTCTGATCATGCTAATGG | CTGCAGTTAGCGTCTCATGTGT |
| ADAM15 | CAGGACGATCTCCCAATTAGC | GGACCAACTCCCTATTCTGTAGC |
| ADAM17 | GTGGATGGTAAAAACGAAAGCG | GGCTAGAACCCTAGAGTCAGG |
| GAPDH | ATGGGGAAGGTGAAGGTCGG | GACGGTGCCATGGAATTTGC |
