# Supplementary material for: CD82 Suppresses ADAM17-Dependent E-Cadherin Cleavage and Cell Migration in Prostate Cancer
Source: Dis Markers. 2020 Nov 1;2020:8899924. doi: 10.1155/2020/8899924 (PMC7654213; doi:10.1155/2020/8899924)
Supplement: Supplementary 2 — Supplementary Table 2: primer sequences used in real-time PCR in the experiments. [file 8899924.f2.docx]

| Target Genes | siRNA sequences（5’—3’） |
| --- | --- |
| NC  CD82 | UUCUCCGAACGUGUCACGUTT  #789：GAAGAGGACAACAGCCUUUTT  #1162：CCCAUCCUGACUGAAAGUATT |
| ADAM9 | #1：CCAGUAUUAUGAUGCUCAA dTdT  #2：CCAGAAUAACAAAGCCUAU dTdT  #3：GCGAAGGAAGUACCUGUAA dTdT |
| ADAM10 | #1：AGACAUUAUGAAGGAUUAU dTdT  #2：GACAUUUCAACCUACGAAU dTdT  #3：GCUAUAGUGACCAGUGUAA dTdT |
| ADAM15 | #1：GCGGGAAUCUGUACACACU dTdT  #2：GCUGGUGACUGGUACUUCA dTdT  #3：GCUGCUUUCUGCGGAAAUA dTdT |
| ADAM17 | #1：GCUUGUUCAUCGAGUGAAA dTdT  #2：GGAUGGUCUAGCAGAAUGU dTdT  #3：GAGAAGCUUGAUUCUUUGC dTdT |

Supplementary TABLE 1: SiRNA sequences used in the experiments.
